# Heterogeneity Within Frailty: Physiological Reserve Phenotypes and Postoperative Recovery After Abdominal Surgery

**DOI:** 10.3390/jcm15031249

**Published:** 2026-02-04

**Authors:** Rafał Cudnik, Luigi Marano, Elena Montanari, Alessandra Marano, Eugenia Semeraro, Mauro Santarelli, Tomasz Cwalinski, Sergii Girnyi, Filippo Luca Fimognari, Virginia Boccardi

**Affiliations:** 1 Emergency Department, “Saint Wojciech” Hospital, “Nicolaus Copernicus” Health Center, 80-530 Gdańsk, Poland; rcudnik@copernicus.gda.pl; 2 Department of Medicine, Academy of Applied Medical and Social Sciences-AMiSNS (Akademia Medycznych i Spolecznych Nauk Stosowanych), 82-300 Elbląg, Poland; 3 Department of General Surgery and Surgical Oncology, “Saint Wojciech” Hospital, “Nicolaus Copernicus” Health Center, 80-530 Gdańsk, Poland; tcwalinski@copernicus.gda.pl (T.C.); sgirnyi@copernicus.gda.pl (S.G.); 4 Emergency General Surgery Unit, General and Specialistic Surgery Department, Hospital Città della Salute e della Scienza di Torino, 10024 Turin, Italy; emontanari@cittadellasalute.to.it (E.M.); amarano@cittadellasalute.to.it (A.M.); eugenia.semeraro@unito.it (E.S.); msantarelli@cittadellasalute.to.it (M.S.); 5 Multidisciplinary Medical Department, Azienda Ospedaliera “Annunziata-Mariano Santo-S. Barbara”, 87100 Cosenza, Italy; f.fimognari@aocs.it; 6 Section of Gerontology and Geriatrics, Department of Medicine and Surgery, University of Perugia, 06132 Perugia, Italy; virginia.boccardi@unipg.it

**Keywords:** handgrip strength, frailty, physiological reserve, surgical outcomes, length of stay

## Abstract

**Background:** Chronological age inadequately captures biological vulnerability among surgical patients. Frailty and muscle strength reflect physiological reserve, yet their combined contribution to postoperative length of stay (LOS) remains insufficiently explored. **Methods:** We conducted a prospective multicenter observational cohort study including 223 adults undergoing elective abdominal surgery. Frailty was assessed using the Fried phenotype, and admission handgrip strength (HGS) was measured with a calibrated dynamometer. Prolonged LOS was defined as >10 days (75th percentile) and also analyzed continuously using ln(LOS + 1). Multivariable logistic and linear regression models adjusted for age, sex, frailty status, and surgical indication. Patients were additionally stratified into four physiological reserve phenotypes combining frailty and HGS. **Results:** LOS ranged from 0 to 68 days; a total of 48 patients (21.6%) experienced prolonged hospitalization. In multivariable logistic regression, frailty (adjusted OR 3.12, 95% CI 1.72–5.67) and oncologic surgery (adjusted OR 7.63, 95% CI 3.12–18.65) were independently associated with prolonged LOS, whereas chronological age was not. Female sex was associated with lower odds of prolonged LOS (adjusted OR 0.39, 95% CI 0.18–0.87). Similar associations were observed when LOS was analyzed continuously. Physiological reserve phenotyping revealed graded LOS distributions: Fit–Strong patients had the shortest stays (mean 5.5 ± 4.3 days), while Frail–Weak patients experienced the longest and most variable hospitalization. **Conclusions:** Postoperative LOS clusters according to multidimensional physiological reserve rather than chronological age. Integrating frailty and muscle strength identifies clinically meaningful phenotypes that may improve perioperative risk stratification beyond age-based approaches and inform personalized perioperative planning, resource allocation, and patient-centered decision-making across heterogeneous surgical populations in worldwide settings.

## 1. Introduction

The progressive ageing of surgical populations represents one of the most consequential demographic shifts facing contemporary perioperative medicine [1]. Advances in surgical techniques and perioperative care have expanded operative eligibility to increasingly older adults, many of whom present with complex vulnerability profiles that extend beyond chronological age alone [2]. Chronological age per se has repeatedly shown limited discriminative capacity in predicting postoperative outcomes, particularly with respect to recovery trajectories and healthcare utilization. Large observational studies and surgical registries demonstrate that patients of similar age may experience markedly divergent postoperative courses, highlighting the inadequacy of age-based thresholds as stand-alone risk stratifiers [3,4,5].

In response to this limitation, frailty has emerged as a clinically meaningful construct intended to capture diminished physiological reserve and heightened susceptibility to stressors [6,7]. Within surgical cohorts, frailty has been associated with increased rates of postoperative complications, prolonged length of hospital stay (LOS), institutionalization, and mortality [8,9,10,11]. Importantly, frailty assessments have demonstrated prognostic value independent of chronological age and traditional comorbidity indices, particularly in patients undergoing major abdominal surgery [4,11,12], and are increasingly incorporated into preoperative assessment pathways.

Nevertheless, frailty is not a homogeneous condition. Patients meeting frailty criteria often exhibit substantial heterogeneity in physical function, muscle performance, and recovery capacity [13]. This heterogeneity suggests that frailty, when treated as a unidimensional construct, may obscure clinically relevant subgroups and contribute to the wide dispersion of postoperative outcomes observed among frail surgical patients, raising questions about whether frailty alone adequately reflects the biological reserve relevant to surgical stress [14].

In this context, handgrip strength (HGS) has gained recognition as a simple, reproducible, and biologically informative marker of muscle function and global physiological reserve. Lower HGS has been consistently associated with increased risks of disability, hospitalization, and mortality in population-based cohorts [4,11,15,16,17], and with prolonged hospitalization, higher complication rates, and delayed functional recovery in surgical settings [15,16,18,19,20,21,22]. As such, muscle strength may serve as a pragmatic indicator of reserve that complements frailty-based assessments.

Length of hospital stay remains a particularly relevant outcome in perioperative research. Beyond reflecting surgical recovery, LOS captures the cumulative impact of complications, functional decline, discharge readiness, and healthcare system factors [3,23,24,25]. Its markedly right-skewed distribution, with a small proportion of patients accounting for the longest hospitalizations, complicates statistical modeling and supports the use of complementary analytical approaches to characterize postoperative trajectories [26].

Despite growing interest in frailty and muscle strength as independent predictors, relatively few studies have examined their combined contribution to postoperative outcomes [4,16,17,27]. Conceptually, a frail patient with preserved muscle strength may differ substantially from a frail patient with marked weakness, even if both meet conventional frailty criteria. Such differences could translate into distinct recovery patterns, particularly following the physiological stress of elective abdominal surgery. This gap in the literature suggests the need for integrative approaches that move beyond linear risk gradients toward multidimensional characterization of physiological reserve.

Within this framework, physiological reserve phenotyping—integrating frailty status and muscle strength—offers a strategy to explore biological clustering rather than isolated predictors. Rather than assuming a uniform progression of vulnerability, phenotyping may better reflect the multidimensional nature of ageing and resilience, aligning with emerging paradigms in geriatric assessment and precision medicine [28,29].

Against this backdrop, we conducted a prospective, multicenter observational study in patients undergoing elective abdominal surgery to examine the relationship between frailty, handgrip strength, and postoperative LOS. Our objectives were threefold: (i) to assess whether frailty and muscle strength retain independent associations with prolonged hospitalization after adjustment for chronological age and surgical indication; (ii) to evaluate the prognostic contribution of age when treated as a continuous variable across its full observed range; and (iii) to explore how postoperative LOS distributions vary across combined frailty–strength phenotypes. We hypothesized that postoperative LOS would cluster more closely according to multidimensional reserve profiles than along chronological age alone, potentially highlighting clinically relevant heterogeneity within conventional frailty categories.

## 2. Materials and Methods

### 2.1. Study Population

Eligible participants were adults (≥18 years) scheduled for elective abdominal surgical procedures requiring postoperative hospital admission. To reduce heterogeneity unrelated to physiological reserve and postoperative recovery, several categories of patients were excluded. These included individuals undergoing diagnostic or purely exploratory procedures without therapeutic intent, palliative surgical interventions performed with symptom control as the primary goal, day-case or ambulatory procedures not requiring inpatient postoperative care, and emergency operations, where acute physiological derangement and time constraints preclude standardized preoperative assessment. Patients unable to provide informed consent were also excluded.

**Figure 1:**
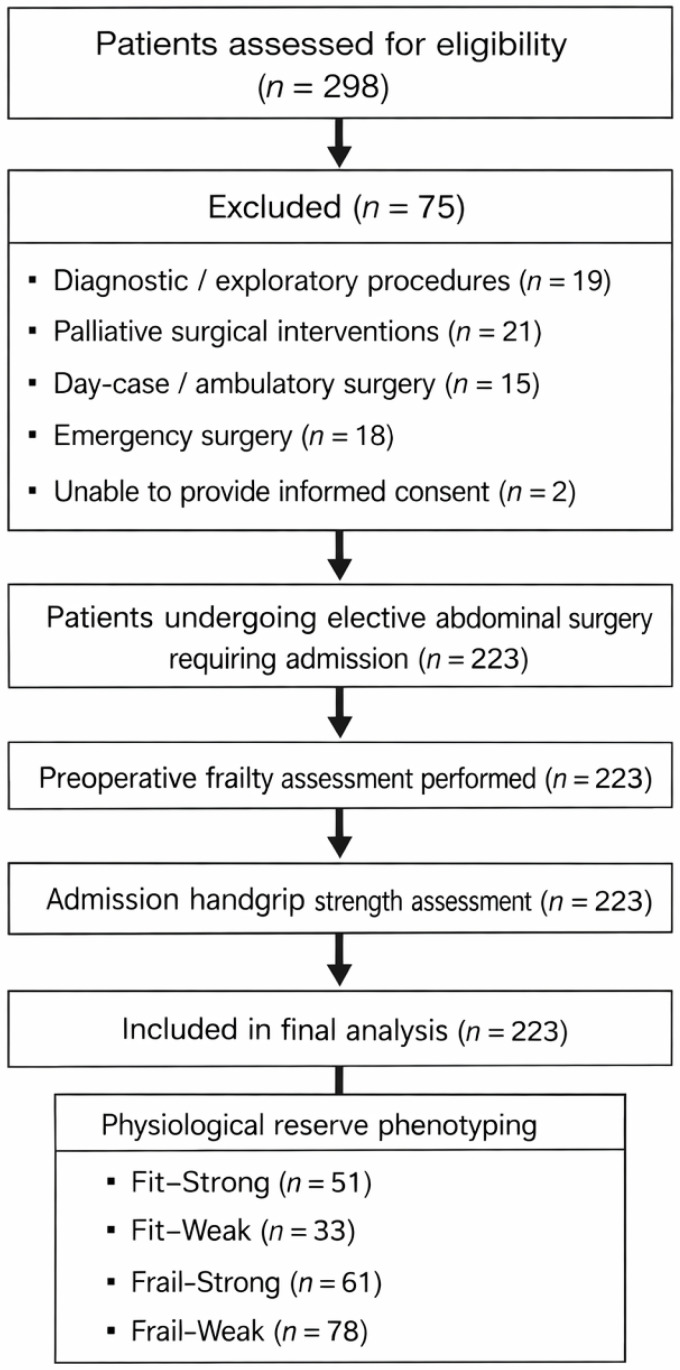
Flow diagram of patient screening, exclusions, and final inclusion. The diagram illustrates the number of patients assessed for eligibility, reasons for exclusion, and the final analytic cohort. All included patients underwent preoperative frailty assessment and admission handgrip strength measurement and were subsequently classified into physiological reserve phenotypes.

Because functional reserve represented a central exposure of interest, the availability of a valid preoperative HGS measurement obtained at hospital admission was required for inclusion in analyses involving muscle strength and physiological reserve phenotyping. No patients were excluded due to missing admission HGS in the present cohort (Figure 1).

### 2.2. Baseline Clinical Variables

Demographic and clinical data were collected prospectively, including age, sex, and surgical indication (oncologic vs. non-oncologic). Classification of procedures as oncologic or non-oncologic was based on the primary surgical indication. Chronological age was analyzed as a continuous variable and summarized using mean, median, and range to reflect the broad age distribution of the cohort. The distribution of elective abdominal surgical procedures, grouped into homogeneous categories according to anatomical site and oncologic intent, is reported in Supplementary Table S1.

### 2.3. Frailty Assessment

Frailty status was assessed preoperatively using the phenotype-based model originally described by Fried et al. [30], focusing on physical and functional vulnerability rather than comorbidity burden. Five criteria were evaluated: unintentional weight loss, self-reported exhaustion, low physical activity, slowness, and muscle weakness. Each criterion contributed one point, yielding a cumulative score ranging from 0 to 5.

Patients were classified as fit (0 criteria), pre-frail (1–2 criteria), or frail (3–5 criteria). For regression analyses and phenotyping, frailty status was treated as a categorical variable (frail vs. fit/non-frail) or combined with muscle strength as described below.

### 2.4. Handgrip Strength Assessment

Handgrip strength was measured at hospital admission using a calibrated digital dynamometer (EH101, Deyard, Shenzhen, China), following standardized procedures in accordance with American Society of Hand Therapists recommendations [31]. Participants were assessed in a seated position with the shoulder adducted, elbow flexed at 90°, and wrist in slight extension. After familiarization, three maximal voluntary isometric contractions were performed with the dominant hand, and the highest recorded value (kg) was retained for analysis.

Admission HGS was analyzed as a continuous variable, dichotomized using sex-specific medians for regression analyses, and combined with frailty status for physiological reserve phenotyping. For descriptive purposes, the overall cohort median is also reported.

### 2.5. Definition of Physiological Reserve Phenotypes

To capture the combined contribution of frailty and muscle strength, patients were stratified into four physiological reserve phenotypes by crossing frailty status with admission HGS dichotomized at the cohort median (23.1 kg): Fit–Strong, Fit–Weak, Frail–Strong, and Frail–Weak. This approach was intended to reflect biological clustering of reserve rather than a linear risk gradient and was used for descriptive and illustrative analyses without causal inference. In addition to median-based dichotomization, a low HGS threshold defined as ≤20th sex-specific percentile was explored in univariable analyses, in line with prior literature.

### 2.6. Outcome Definition

Postoperative LOS was defined as the number of days from surgery to hospital discharge. LOS exhibited a markedly right-skewed distribution and was analyzed in two complementary ways. First, prolonged LOS was defined a priori as LOS > 10 days, corresponding to the 75th percentile of the cohort distribution [32,33,34]. Second, LOS was analyzed as a continuous variable using log-transformed values [ln(LOS + 1)] to accommodate skewness and retain information across the full observed range.

### 2.7. Study Design and Ethical Approval

This prospective, multicenter observational cohort study was designed to examine perioperative vulnerability through functional and physiological stratification rather than chronological age alone. The study project was initiated in January 2023, including protocol development and coordination among participating centers. Consecutive adult patients undergoing elective abdominal surgery were prospectively enrolled after ethics committee approval (27 February 2023), from February 2023 to June 2025, at participating academic surgical centers. The study was conducted in accordance with the Declaration of Helsinki and Good Clinical Practice guidelines. Ethical approval was obtained from the coordinating institutional ethics committee of the Hospital Città della Salute e della Scienza di Torino (protocol code 0025277, approved on 27 February 2023). All participants provided written informed consent prior to inclusion. Reporting adheres to the Strengthening The Reporting of Observational Studies in Epidemiology (STROBE) statement for observational studies [35]. This observational study did not include an external control group. All comparisons were performed within the study cohort, with non-frail patients and those with higher handgrip strength serving as internal reference groups for regression and phenotyping analyses.

### 2.8. Statistical Analysis

All analyses were performed using IBM SPSS Statistics version 26 (IBM Corp., Chicago, IL, USA). Figures were generated using GraphPad Prism version 10.6.1. Continuous variables are reported as mean ± standard deviation, median with interquartile range (IQR), and range, as appropriate. Categorical variables are reported as counts and percentages.

Univariable logistic regression models were used to examine crude associations between candidate predictors (age, sex, frailty status, admission HGS, surgical indication) and prolonged LOS. Odds ratios (ORs) with 95% confidence intervals (CI) are reported. A multivariable logistic regression model was specified a priori, including age (continuous, per year), sex, frailty status was entered as a binary variable (frail [≥3 criteria] vs. fit/pre-frail [0–2 criteria]), and surgical indication (oncologic vs. non-oncologic). Given the conceptual overlap between frailty and muscle weakness, admission HGS was not included in the primary multivariable model; however, an additional sensitivity model including HGS was performed and is reported in Supplementary Table S2. Model performance was evaluated using the omnibus likelihood ratio test, Nagelkerke R^2^, and Hosmer–Lemeshow goodness-of-fit test. Discrimination was assessed using receiver operating characteristic (ROC) curve analysis, with area under the curve (AUC) and 95% CI reported.

The linearity of the logit for age was formally tested using the Box–Tidwell approach, and multicollinearity was assessed using variance inflation factors (VIFs). To verify that findings were not driven by outcome dichotomization, LOS was additionally analyzed as a continuous variable using multivariable linear regression with ln(LOS + 1) as the dependent variable. Regression coefficients (B), 95% CI, adjusted R^2^, and model diagnostics are reported.

LOS distributions across physiological reserve phenotypes were summarized descriptively using mean ± SD, median (IQR), and range. These analyses were explicitly descriptive and intended to illustrate biological clustering rather than to test hypotheses.

An a priori sample size calculation was performed using G*Power version 3.1.9.7 (University of Düsseldorf, Düsseldorf, Germany). Assuming a small-to-moderate effect size (f^2^ = 0.10), a two-sided α level of 0.05, and 80% power, a minimum of 179 participants was required for multivariable regression analyses including up to four predictors. The final sample of 223 patients exceeded this threshold.

All statistical tests were two-sided, with *p* < 0.05 considered statistically significant. P-values were interpreted cautiously, with emphasis placed on effect size patterns and consistency across analytical approaches.

## 3. Results

### 3.1. Study Population Characteristics

The study cohort comprised 223 consecutive patients undergoing surgical procedures. Chronological age, analyzed as a continuous variable, showed a wide distribution (range: 19–93 years), underscoring the heterogeneity of the population. Postoperative length of stay (LOS) demonstrated a markedly right-skewed distribution, with values ranging from 0 to 68 days, reflecting diverse postoperative recovery trajectories.

Using the 75th percentile as a clinically meaningful threshold, 48 patients (21.6%) experienced prolonged hospitalization, defined as LOS > 10 days, while 174 patients (78.4%) were discharged within 10 days.

Preoperative frailty status was systematically assessed in all patients. Admission HGS was available for the entire cohort and showed substantial interindividual variability, with values ranging from 4.0 to 46.0 kg. The median admission HGS was 23.1 kg, with a near-equal distribution of patients above (112 patients, 50.2%) and below (111 patients, 49.8%) this value (Table 1).

### 3.2. Univariable Associations with Prolonged Length of Stay

In univariable logistic regression analyses, prolonged LOS appeared to cluster around indicators of physiological vulnerability and surgical complexity. Frailty status was significantly associated with extended hospitalization, with frail patients exhibiting more than a twofold increase in the odds of LOS exceeding 10 days (*p* < 0.001). Similarly, low admission HGS, defined as ≤20th sex-specific percentile (used for risk association analyses), was associated with increased odds of prolonged LOS (odds ratio approximately 2.2, *p* = 0.034).

The strongest univariable association was observed for oncologic surgery, which markedly increased the probability of extended hospitalization (odds ratio approximately 7.4, *p* < 0.001). By contrast, chronological age, when treated as a continuous variable across its full range, showed only a borderline association with prolonged LOS (*p* ≈ 0.06), while sexwas not significantly associated with the outcome in unadjusted analyses (*p* > 0.30).

### 3.3. Multivariable Predictors of Prolonged Hospitalization

A multivariable logistic regression model including age, sex, frailty status, and oncologic versus non-oncologic indication demonstrated good overall performance (Omnibus χ^2^ = 48.1, *p* < 0.001). The model explained a substantial proportion of outcome variability (Nagelkerke R^2^ = 0.30) and showed acceptable calibration (Hosmer–Lemeshow χ^2^ = 14.0, *p* = 0.083).

After adjustment, frailty status remained independently associated with prolonged LOS (adjusted OR 3.12, 95% CI 1.72–5.67, *p* < 0.001). Oncologic surgery emerged as the strongest independent determinant (adjusted OR 7.63, 95% CI 3.12–18.65, *p* < 0.001). Female sex was independently associated with lower odds of prolonged hospitalization (adjusted OR 0.39, 95% CI 0.18–0.87, *p* = 0.016).

In contrast, chronological age, across its observed range, did not retain an independent association with prolonged LOS (adjusted OR 0.99 per year, *p* = 0.535), suggesting limited incremental prognostic value once frailty and surgical context were considered (Table 2). A sensitivity analysis including admission HGS in the multivariable model is provided in Supplementary Table S2.

### 3.4. Assessment of Age Linearity

The assumption of linearity in the logit for age was formally evaluated using the Box–Tidwell approach. The interaction term between age and its natural logarithm was not statistically significant (*p* = 0.393), indicating no detectable deviation from linearity across the age range observed in the cohort. Age was therefore retained as a continuous linear predictor in all models.

### 3.5. Discriminative Performance of the Multivariable Model

Model discrimination was assessed using receiver operating characteristic (ROC) curve analysis based on predicted probabilities derived from the multivariable logistic regression model. The model demonstrated good discriminative ability, with an area under the curve (AUC) of 0.81 (95% CI 0.75–0.87, *p* < 0.001), indicating effective separation between patients with prolonged and standard postoperative hospital stays.

### 3.6. Continuous Analysis of Length of Stay

To assess whether findings were influenced by the dichotomization of LOS, the outcome was additionally analyzed as a continuous variable using log-transformed LOS [ln(LOS + 1)], accommodating the observed LOS range of 0–68 days. In linear regression analysis including the same covariates, the model remained statistically significant (adjusted R^2^ ≈ 0.34, *p* < 0.001).

Consistent with the logistic analysis, frailty status and oncologic surgery were independently associated with longer LOS (*p* < 0.001 for both). Female sex was associated with shorter LOS (*p* = 0.020), whereas chronological age again showed no independent association (*p* = 0.269) (Table 3). Collinearity diagnostics were acceptable (VIF range 1.04–1.39), and inspection of residuals supported the appropriateness of the log-transformed model.

### 3.7. Physiological Reserve Phenotyping

To explore the combined contribution of frailty and muscle strength, patients were stratified into four physiological reserve phenotypes based on frailty status and admission HGS (median split at 23.1 kg):

- Fit–Strong: 51 patients (22.9%);
- Fit–Weak: 33 patients (14.8%);
- Frail–Strong: 61 patients (27.4%);
- Frail–Weak: 78 patients (35.0%).

All phenotypes were adequately represented.

### 3.8. Length of Stay Across Physiological Reserve Phenotypes

Clear differences in LOS distribution were observed across physiological reserve phenotypes. Fit–Strong patients exhibited the shortest and least variable hospital stays, with LOS ranging from 1 to 25 days, a mean of 5.5 ± 4.3 days, a median of 5.0 days, and an interquartile range (IQR) of 5 days.

Fit–Weak patients showed a similar mean LOS (5.5 ± 7.0 days), but a wider range (2–40 days), a shorter median (3.0 days), and increased dispersion (IQR 4 days), suggesting heterogeneous recovery despite preserved frailty status.

**Figure 2:**
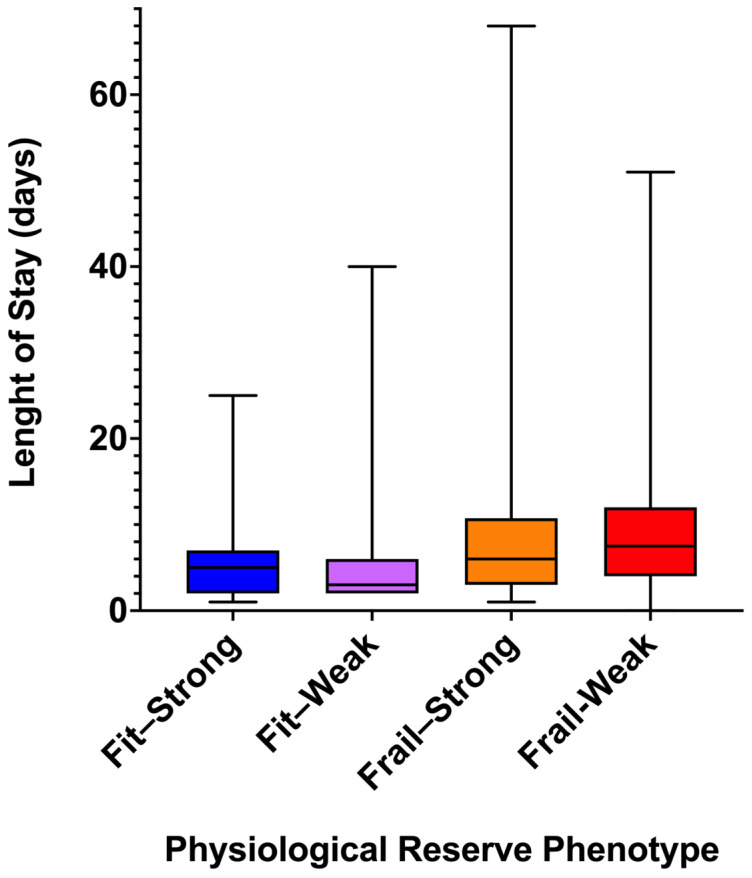
Length of stay across physiological reserve phenotypes. Box-and-whisker plots illustrate the distribution of postoperative length of stay (LOS) according to physiological reserve phenotypes defined by frailty status and admission handgrip strength (HGS) dichotomized using sex-specific median values. Boxes represent the interquartile range, the horizontal line indicates the median, and whiskers extend to minimum and maximum values. No statistical comparisons are shown.

Among frail patients, LOS was consistently longer. Frail–Strong individuals had the broadest LOS range (1–68 days), a mean of 9.4 ± 10.8 days, a median of 6.0 days, and an IQR of 7.75 days, indicating substantial variability. Frail–Weak patients experienced uniformly prolonged hospitalization, with LOS ranging from 0 to 51 days, a mean of 9.1 ± 8.0 days, a median of 7.5 days, and an IQR of 8 days (Table 4 and Figure 2).

Overall, these patterns suggest that postoperative LOS clusters according to combined frailty–strength phenotypes, rather than along a single dimension of vulnerability. These comparisons are descriptive in nature, and no formal statistical testing was performed across physiological reserve phenotypes.

## 4. Discussion

In this prospective multicenter cohort of patients undergoing elective abdominal surgery, we observed that postoperative length of stay aligned more closely with indicators of physiological reserve than with chronological age. Frailty status and surgical indication emerged as the principal determinants of prolonged hospitalization, whereas age—modeled as a continuous variable across its full observed range—did not retain independent prognostic significance once these factors were accounted for. Taken together, these findings suggest that biological vulnerability, rather than age alone, is likely more informative in shaping postoperative recovery trajectories.

Muscle strength further nuanced this association. Although low admission handgrip strength was associated with prolonged LOS in univariable analyses, its clinical relevance became most apparent when interpreted alongside frailty status. When combined into physiological reserve phenotypes, clear and consistent gradients in LOS distribution emerged. Patients classified as Fit–Strong experienced the shortest and least variable hospital stays, while Frail–Weak patients showed uniformly prolonged and dispersed LOS. Intermediate phenotypes—Fit–Weak and Frail–Strong—displayed overlapping yet distinct recovery profiles, suggesting that neither frailty nor muscle strength in isolation fully captures postoperative resilience. In practical terms, our findings may indicate that how vulnerability is expressed matters at least as much as whether it is present.

These observations are concordant with the growing literature questioning the primacy of chronological age in perioperative risk stratification [5,36,37]. Multiple studies have shown that age explains only a limited proportion of variance in postoperative outcomes, particularly once functional and frailty-related variables are introduced into predictive models [38,39]. The absence of an independent age effect in our adjusted analyses reinforces this perspective and supports the view that age-based thresholds alone may be insufficient for contemporary surgical decision-making.

The association between frailty and prolonged LOS observed in our cohort is consistent with prior surgical and geriatric studies linking frailty to adverse postoperative outcomes [14,40,41,42,43,44,45,46]. At the same time, our data highlight substantial heterogeneity within frail patients. In particular, the wide dispersion of LOS among frail individuals—especially those with preserved muscle strength—suggests that frailty, when treated as a single construct, may obscure clinically relevant differences in recovery potential.

Handgrip strength has been extensively validated as a prognostic marker in ageing research, with lower values associated with increased risks of disability, hospitalization, and mortality [15,16,27,47]. In surgical populations, reduced preoperative HGS has been linked to longer LOS and impaired postoperative recovery [4,17,18,20,48]. Our findings are consistent with these reports but also extend them by suggesting that the prognostic meaning of HGS is context-dependent. When considered alone, HGS identifies vulnerability; when integrated with frailty status, it appears to delineate biologically distinct reserve profiles with differing recovery trajectories.

The phenotype-based approach adopted in this study reflects a shift away from linear risk modeling toward biological clustering. Rather than assuming a uniform gradient of vulnerability, physiological reserve phenotyping acknowledges that ageing-related decline unfolds across multiple, partially independent domains [29,41,49,50,51]. Similar conceptual frameworks have been proposed in geriatric medicine and precision ageing research, emphasizing intrinsic capacity and functional reserve over isolated risk factors [1,14,28,38].

Notably, the Frail–Strong phenotype observed in our cohort challenges the assumption that frailty uniformly implies poor functional reserve [6,10,39,40,43,52,53,54,55]. These patients exhibited LOS distributions intermediate between Fit–Weak and Frail–Weak groups, which may suggest that preserved muscle strength partially mitigates the clinical impact of frailty-related deficits. Conversely, Fit–Weak patients—despite not meeting frailty criteria—showed heterogeneous and occasionally prolonged hospital stays, indicating that muscle weakness alone may represent an early or under-recognized manifestation of vulnerability.

From a clinical perspective, these findings support the routine assessment of both frailty and muscle strength in the preoperative setting using feasible, low-cost tools [4]. Phenotype-based stratification may help identify patients who appear similar by age or frailty category but differ substantially in recovery potential, with implications for perioperative counseling, discharge planning, and allocation of supportive resources. In particular, patients classified as Fit–Weak or Frail–Strong may represent targets for tailored interventions, such as prehabilitation or enhanced postoperative support.

At a health-system level, LOS remains a critical outcome reflecting not only surgical recovery but also complication burden, functional decline, and resource utilization [4,17,23,26]. The ability to anticipate prolonged hospitalization through reserve-based phenotypes could assist in optimizing perioperative pathways and aligning care intensity with biological risk rather than chronological age alone.

Several limitations should be acknowledged. Although the cohort was prospectively collected, the observational design precludes causal inference. Physiological reserve phenotypes were derived using median-based dichotomization of handgrip strength, a pragmatic approach that may limit external generalizability to populations with different strength distributions. LOS is also influenced by institutional practices, discharge policies, and social determinants that were not fully captured in this analysis. In addition, postoperative complications were not the primary focus of the present study and may partially mediate the observed associations between reserve profiles and LOS. Finally, external validation in independent cohorts will be essential before phenotype-based approaches can be widely implemented.

## 5. Future Directions

Future studies should seek to validate physiological reserve phenotypes across diverse surgical populations and healthcare settings, ideally using standardized strength thresholds and longitudinal functional outcomes. Incorporation of additional biological and functional markers, such as inflammatory parameters, nutritional status, or mobility assessments, may further refine reserve characterization. Interventional research is also needed to determine whether modifying components of physiological reserve can translate into meaningful improvements in postoperative recovery.

## 6. Conclusions

In summary, our findings suggest that postoperative length of stay following elective abdominal surgery aligns more closely with multidimensional measures of physiological reserve than with chronological age alone. By integrating frailty status and handgrip strength into phenotype-based profiles, we demonstrate clinically meaningful heterogeneity in postoperative recovery trajectories within conventional risk categories. In particular, patients who appear similar when classified by age or frailty alone may experience substantially different lengths of hospitalization when muscle strength is taken into account.

This reserve-oriented perspective reinforces the limitations of age-based stratification and supports a more nuanced approach to perioperative assessment that incorporates functional and biological vulnerability. From a clinical standpoint, combining frailty assessment with a simple measure of muscle strength may improve the identification of patients at risk for prolonged hospitalization and facilitate more individualized perioperative planning, resource allocation, and discharge strategies. In sensitivity analyses, admission handgrip strength did not retain an independent association with prolonged length of stay once frailty status was accounted for, supporting its interpretation as a component of physiological reserve phenotyping rather than as a standalone predictor.

Although observational in nature, this study contributes to a growing body of evidence emphasizing the importance of physiological reserve in surgical outcomes and provides a framework for future validation and interventional research. Integrating multidimensional reserve assessment into routine surgical practice may represent a step toward more biologically informed and patient-centered perioperative care.

## Figures and Tables

**Table 1:** Baseline characteristics of the study population (n= 223).

Variable	Value
**Age, years**	
Mean ± SD	64.7 ± 15.1
Median	65
Range	19–93
**Sex, n (%)**	
Female	102 (45.7)
Male	121 (54.3)
**Frailty status, n (%)**	
Fit/non-frail	84 (37.7)
Frail	139 (62.3)
**Admission handgrip strength (kg)**	
Median	23.1
≤median (≤23.1 kg), n (%)	111 (49.8)
>median (>23.1 kg), n (%)	112 (50.2)
Range	4.0–46.0
**Surgical indication, n (%)**	
Oncologic surgery	118 (52.9)
Non-oncologic surgery	105 (47.1)
**Length of stay (LOS), days**	
Range	0–68
Prolonged LOS (>10 days), n (%)	48 (21.6)
**Physiological reserve phenotype, n (%)**	
Fit–Strong	51 (22.9)
Fit–Weak	33 (14.8)
Frail–Strong	61 (27.4)
Frail–Weak	78 (35.0)

Data are presented as mean ± SD, median, or number (percentage), as appropriate. Percentages may not sum to 100 due to rounding.

**Table 2:** Univariable and multivariable logistic regression analyses for prolonged length of stay (>10 days).

Variable	Univariable OR (95% CI)	*p* Value	Multivariable OR (95% CI)	*p* Value
Age (per 1-year increase)	0.99 (0.96–1.02)	0.060	0.99 (0.96–1.02)	0.535
Female sex (vs. male)	0.73 (0.38–1.38)	0.318	0.39 (0.18–0.87)	0.016
Frailty status (frail vs. fit)	3.12 (1.72–5.67)	<0.001	3.12 (1.72–5.67)	<0.001
Lower admission HGS (sex-specific median)	2.20 (1.06–4.58)	0.034	—	—
Oncologic surgery (vs. non-oncologic)	7.63 (3.12–18.65)	<0.001	7.63 (3.12–18.65)	<0.001

“—” indicates variables not retained in the multivariable model.

**Table 3:** Multivariable linear regression analysis for log-transformed length of stay [ln(LOS + 1)].

Variable	B Coefficient	95% Confidence Interval	*p* Value
Age (per 1-year increase)	−0.01	−0.03 to 0.02	0.269
Female sex (vs. male)	−0.32	−0.59 to −0.05	0.020
Frailty status (frail vs. fit)	0.74	0.43 to 1.05	<0.001
Oncologic surgery (vs. non-oncologic)	1.12	0.78 to 1.46	<0.001

Model statistics: Adjusted R^2^ = 0.34; Overall model *p* < 0.001; Variance inflation factor (VIF) range: 1.04–1.39. Linear regression was performed using log-transformed length of stay [ln(LOS + 1)] to account for right-skewed LOS distribution. B coefficients represent the expected change in ln(LOS + 1) per unit increase in the predictor. Age was analyzed as a continuous variable. All variables shown were entered simultaneously into the model.

**Table 4:** Length of stay according to physiological reserve phenotypes.

Physiological Reserve Phenotype	N (%)	Mean LOS ± SD (Days)	Median LOS (IQR), Days	Range (Days)
Fit–Strong	51 (22.9%)	5.5 ± 4.3	5.0 (5.0)	1–25
Fit–Weak	33 (14.8%)	5.5 ± 7.0	3.0 (4.0)	2–40
Frail–Strong	61 (27.4%)	9.4 ± 10.8	6.0 (7.75)	1–68
Frail–Weak	78 (35.0%)	9.1 ± 8.0	7.5 (8.0)	0–51

Physiological reserve phenotypes were defined by combining frailty status and admission handgrip strength (HGS) using the cohort median value (23.1 kg). Length of stay (LOS) is reported as mean ± SD, median (interquartile range), and range.

## Data Availability

The data supporting the findings of this study are available from the corresponding author upon reasonable request. The data are not publicly available due to privacy and ethical restrictions related to the use of clinical and patient-level information.

## Supplementary Materials

The following supporting information can be downloaded at: https://www.mdpi.com/article/10.3390/jcm15031249/s1, Table S1: Elective abdominal surgical procedures included in the study cohort (N = 223); Table S2: Sensitivity multivariable logistic regression analysis for prolonged length of stay (>10 days), including admission handgrip strength.

## Abbreviations

The following abbreviations are used in this manuscript:

LOS	Length of stay
HGS	Handgrip strength
