# Supplementary material for: Heterogeneity Within Frailty: Physiological Reserve Phenotypes and Postoperative Recovery After Abdominal Surgery
Source: J Clin Med. 2026 Feb 4;15(3):1249. doi: 10.3390/jcm15031249 (PMC12898188; doi:10.3390/jcm15031249)
Supplement: Supplementary file 1 [file jcm-15-01249-s001.zip › jcm-4108289-supplementary.pdf]

Journal of Clinical Medicine

MDPI

Supplementary Table S1. Elective abdominal surgical procedures included in the study cohort (N = 223)

Surgical category	Representative procedures included	N (%)
Colorectal surgery	Right/left hemicolectomy (open, laparoscopic, robotic), sigmoidectomy, colectomy, anterior rectal resection (RAR) ± ileostomy/colostomy	78 (35.0%)
Gastric surgery	Partial, subtotal, and total gastrectomy ± D2 lymphadenectomy; gastric wedge resection; gastroenterostomy	42 (18.8%)
Hepatopancreatobiliary surgery	Distal pancreatectomy, pancreatic head resection, splenectomy, partial hepatectomy, pancreatic enucleation	24 (10.8%)
Peritoneal surface malignancy	Cytoreductive surgery ± HIPEC; PIPAC	19 (8.5%)
Stoma-related procedures	Ileostomy or colostomy creation, closure, or recanalization	21 (9.4%)
Abdominal wall surgery	Umbilical, epigastric, and incisional hernia repair	27 (12.1%)
Biliary surgery	Cholecystectomy (isolated or combined with other elective abdominal procedures)	32 (14.3%)
Gynecologic oncologic surgery	Hystero-annessiectomy ± omentectomy, peritonectomy, pelvic resections	14 (6.3%)
Other elective abdominal surgery	Appendectomy, adhesiolysis, retroperitoneal mass excision, neuroendocrine tumor resections	16 (7.2%)

All procedures were elective abdominal surgeries performed with therapeutic intent and requiring postoperative hospital admission. *HIPEC*, hyperthermic intraperitoneal chemotherapy; *PIPAC*, pressurized intraperitoneal aerosol chemotherapy.

Supplementary Table S2. Sensitivity multivariable logistic regression analysis for prolonged length of stay (>10 days), including admission handgrip strength

Outcome: Prolonged postoperative length of stay (LOS > 10 days)

**Variable**	**Adjusted OR**	**95% CI**	**p value**
Age (per 1-year increase)	1.00	0.97 – 1.03	0.950
Female sex (vs male)	3.69	1.45 – 9.39	0.006
Frailty status (frail vs fit)	0.35	0.15 – 0.84	0.018
Admission handgrip strength (strong vs weak)	2.76	1.09 – 7.00	0.033
Oncologic surgery (vs non-oncologic)	0.12	0.05 – 0.30	<0.001

Model statistics

- Number of observations: **223**
- Omnibus test of model coefficients: $\chi^2 = 42.9$, $p < 0.001$
- Nagelkerke R^2 = **0.27**
- Cut-off probability = 0.50

Odds ratios (ORs) were derived from a multivariable logistic regression model including age, sex, frailty status, admission handgrip strength (HGS), and surgical indication.

Reference categories: male sex, fit (non-frail), weak admission HGS, non-oncologic surgery.

Admission HGS was dichotomized using sex-specific median values.

This sensitivity analysis was performed to evaluate the independent contribution of admission HGS beyond frailty status.
